# SPONTANEOUS RUPTURE OF BAKER’S CYST - CASE SERIES

**DOI:** 10.1590/1413-785220253302e290230

**Published:** 2025-10-13

**Authors:** GILBERTO LUIS CAMANHO, RICCARDO GOMES GOBBI, MÁRCIA UCHÔA DE REZENDE, GUILHERME PEREIRA OCAMPOS

**Affiliations:** 1. Universidade de Sao Paulo, Faculdade de Medicina, Hospital das Clinicas (HC-FMUSP), Instituto de Ortopedia e Traumatologia, Sao Paulo, SP, Brazil.

**Keywords:** Popliteal Cyst, Acute Pain, Joint Capsule, Synovial Membrane, Synovial Fluid, Osteoarthritis, Cisto Popliteal, Dor Aguda, Cápsula Articular, Membrana Sinovial, Líquido Sinovial, Osteoartrite

## Abstract

**Objective:**

The presence of popliteal cysts is common, although often unkown to the patient. When spontaneous rupture occurs, the clinical presentation is typically dramatic, frequently prompting the patient to seek emergency care. Eventually the condition can be mistaken for other pathologies, more commonly deep vein thrombosis, leading to inappropriate treatment. The objective of this study is to describe a case series of spontaneous Baker’s cyst ruptures, with a 2-year follow-up.

**Methods:**

Sixteen consecutive patients with symptomatic ruptured popliteal cyst were treated acutely with anti-inflammatory measures and physical therapy.

**Results:**

All patients had a good resolution of their cases with conservative treatment over an average period of one week, and there was no recurrence of cyst rupture.

**Conclusion:**

Spontaneous rupture of Baker’s cyst in adult patients can be very symptomatic and disabling; however, once diagnosed, it responds well to conservative treatment and rarely recurs. Level of Evidence IV; Case Series.

## RESUMO

**Objetivo:**

A presença de cistos poplíteos é comum, embora nem sempre seja de conhecimento do paciente. Quando ocorre a ruptura espontânea do cisto, o paciente apresenta um quadro exuberante, geralmente buscando assistência no pronto-socorro. Eventualmente, a condição pode ser confundida com outras patologias, mais frequentemente com a trombose venosa profunda, o que leva a um tratamento inadequado. O objetivo é descrever uma série de casos de ruptura espontânea de cistos de Baker, com acompanhamento de 2 anos.

**Métodos:**

Dezesseis pacientes consecutivos com ruptura sintomática de cisto poplíteo foram tratados agudamente com medidas anti-inflamatórias e fisioterapia.

**Resultados:**

Todos os pacientes apresentaram uma boa resolução de seus quadros com tratamento conservador por um período médio de uma semana, e não houve recorrência da ruptura do cisto.

**Conclusão:**

A ruptura espontânea do cisto de Baker em pacientes adultos pode ser muito sintomática e incapacitante, mas uma vez diagnosticada, evolui bem com tratamento conservador e raramente recorre. Nível de Evidência IV; Série de Casos.

## INTRODUCTION

The presence of cysts in the posterior region of the knee is a common finding in the evaluation of patients with problems in this joint.^1^

Asymptomatic cysts in adult patients result from some irritative process of traumatic or inflammatory origin in the synovial tissue resulting in an increased production of synovial fluid.^2,3^ Through a communication in the posteromedial region of the joint capsule, the liquid leaks into the bursa between the medial and semimembranous gastrocnemius tendons, and through a valve mechanism a cyst is formed, called Baker’s cyst,^4,5^ described by William Baker in 1877.^6^

Acute rupture of Baker’s cyst (RABC) causes a very symptomatic condition, which occurs in patients who, in general, are unaware that they have the cysts. The intensity of the symptoms, which appear acutely, frightens patients and medical colleagues who are not familiar with the condition, a fact that often leads to inappropriate behavior.

Despite its clinical importance, it is a topic that is little reported and studied. The objective of this work is to describe the symptoms, treatment and follow-up of RABC.

## METHODS

Retrospective case series with 16 patients who presented with RABC. Casuistry originating from the project approved by the ethics committee 66509923.5.0000.0068.

Included were:

- Adult patients diagnosed with RABC, confirmed by clinical examination and ultrasound or MRI;
- Minimum of two years of follow-up;
- Absence of history of trauma at the time of symptom onset;
- Intensity of the condition sufficient to seek medical help within seven days at the onset of the condition.

The medical records were searched to obtain: age, sex, laterality, previous pathology in the knee, symptoms, initial diagnosis given in the first medical evaluation, diagnosis of joint pathology related to the origin of the cyst, treatment instituted, and results after two years of follow-up.

## RESULTS

Among the 16 patients included with RABC, 10 were male (62.5%). The right side was affected nine times (56.2%). The average age was 59.4 years (±7.08), varying between 46 years and 71 years.

**Figure 1:**
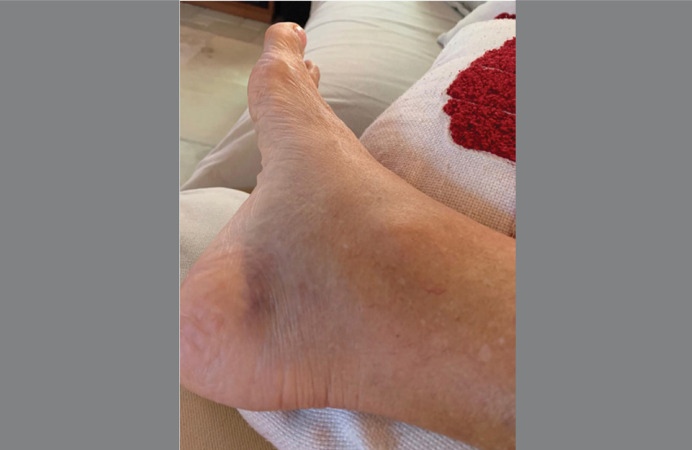
Hematoma on the medial aspect of the ankle in a patient with spontaneous rupture of a Baker's cyst.

Source: Author's personal fie.

All patients reported sudden and disabling pain in the posterior region of the knee, with worsening on palpation of the posterior region of the knee and calf, limitation of ankle dorsiflexion due to pain and lameness. The pain was progressive in the first few hours and was not sensitive to common analgesics, leading patients to emergency care. Its onset was not related to any traumatic event. In three cases with more than five days of evolution, distal hematomas were found on the posterior aspect of the leg or on the medial aspect of the ankle (Figure 1).

**Figure 2:**
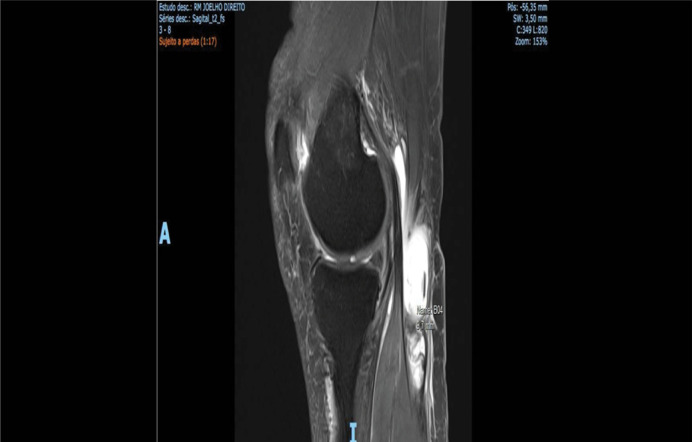
Sagittal magnetic resonance image demonstrating a partially ruptured Baker's cyst in its distal part.

Source: Author's personal fie.

RABC was initially characterized in 11 cases by ultrasound, and in five by MRI (Figure 2). The differential diagnosis was deep vein thrombosis in most cases.

No patient related the symptom to previous pathology in their knee, although 12 were aware of some pathology in the affected knee. The diagnoses of related joint pathologies are detailed in Table 1.

**Table 1:** Distribution of Previous Joint Pathologies in Patients with RABC.

	Number of Cases	Percentage
Knee Osteoarthritis	6	37.5%
Meniscal Injury	4	25%
Previous Arthroplasty	2	12.5%
Previous Ligament Surgery	2	12.5%
Synovial Pathology	2	12.5%

RABC: acute rupture of Baker’s cyst.

Among the four patients who were unaware of having any knee problems, two were diagnosed with osteoarthritis and two with meniscal injuries.

Treatment was always initially non-surgical with rest, ice and NSAIDs for five to seven days. After the acute phase, everyone underwent physiotherapy for analgesia and muscle rehabilitation until symptoms resolved.

The evaluation of patients after two years showed that all patients had a good evolution with conservative treatment for a period of up to three weeks, and there was no recurrence in any case.

Only one of those with a meniscal injury underwent partial medial meniscectomy before two years of follow-up due to persistence of joint and posterior knee symptoms.

## DISCUSSION

The presence of popliteal cysts depends on the population studied and the technique used for diagnosis. In studies of asymptomatic knees in adults, popliteal cysts have been identified in 4.7% to 37% of cases.^2,3^

The integrity of the joint capsule decreases with age, and one theory is that the opening that forms the cyst results from an injury to the joint capsule degenerate.^4,5^ Rauschning^7^ noted that when no opening was found, capsular thinning allows herniation of synovial tissue.

When intact, it is visible in some cases, but most of the time the diagnosis results from an imaging exam, with ultrasound and resonance being the most common.^8^

In most cases it is asymptomatic and goes unnoticed by the patient. The treatment is based on the basic pathology that led to the cyst, surgical approach to the cyst is only necessary in cases of compression of important structures in the posterior region of the knee, a very rare occurrence.

The surgical approach to cyst resection leads to a high rate of recurrence and is not recommended.^7^ A recent meta-analysis^9^ on Baker’s cyst treatment techniques compared the increase in communication between the cyst and the joint cavity and the closure of this communication. The success rates were 96.7% and 84.6% in the communication expansion group and the communication closure group, respectively. Resection of the cyst wall or non-resection of the wall has success rates of 98.2% and 94.7%, respectively.

RABC is uncommon and treated with some disregard in the literature, most of the time as case reports,^10^ but it must be remembered due to the importance of the intensity of the initial symptoms, which when treated without knowledge lead to dramatic situations.^11^ We have already followed a case of a patient whose RABC was diagnosed as compartment syndrome and underwent fasciotomy, causing unnecessary suffering to the patient.

We believe that asymptomatic cyst ruptures occur, as it is not uncommon to notice images on MRI described as ruptured cysts by the radiologist, without clinical correspondence.

Conservative initial treatment brings good results. Assessment of the cause of the cyst should guide the follow-up sequence. We had four patients with knee osteoarthritis without synovitis. Everyone had already taken some medication due to knee discomfort. Two of them needed of total arthroplasty in the evolution of symptoms. Four patients had degenerative medial meniscal injuries with involvement of the articular cartilage. One patient underwent meniscectomy shortly after RABC. It progressed well, without recurrence.

Some patients had previous knee surgery. Two patients had total arthroplasty for knee arthrosis, carried out three and five years ago. They had no complaints about the affected knee despite having RABC. Two patients had undergone successful anterior cruciate ligament reconstruction and had no other apparent cause for the cyst. Everyone progressed well after treatment.

Two patients were being treated for inflammatory synovial disease.

RABC treatment was conservative, as described, in all cases and resulted in a good evolution without recurrence.

## CONCLUSION

Spontaneous rupture of a Baker’s cyst in adult patients can be very symptomatic and disabling, but once diagnosed, it progresses well with conservative treatment and rarely recurs.
